# Plyometric Performance in U13 Basketball: Influence of Modified Competitions and Maturational Status with GPS Tracking

**DOI:** 10.3390/s26020552

**Published:** 2026-01-14

**Authors:** Ricardo André Birrento Aguiar, Francisco Javier García-Angulo, Riccardo Izzo, Enrique Ortega-Toro

**Affiliations:** 1 Faculty of Sports Sciences, University of Murcia, 30720 Santiago de la Ribera, Spain; ra.birrentoaguiar@um.es (R.A.B.A.); eortega@um.es (E.O.-T.); 2 Human Movement and Sports Science (HUMSE), Faculty of Sports Sciences, University of Murcia, 30720 Santiago de la Ribera, Spain; 3 Department of Biomolecular Sciences, School of Sport and Health Sciences, University of Urbino Carlo Bo, 61029 Urbino, Italy; riccardo.izzo@uniurb.it

**Keywords:** biological maturation, plyometric performance, youth basketball

## Abstract

The aim of this study was to analyze the effects of different competition formats on the plyometric performance of under-13 basketball players, considering the influence of maturational age and monitored through GPS devices. Thirty-seven under-13 male basketball players (age = 12.91 ± 0.57 years) from four southeast Spanish teams participated in two different tournaments. On the first day, the tournament was played according to the official Spanish Basketball Federation (FEB) rules for under-14 players. On the second day, the competition was held with modified rules (Modified Tournament), in which the basket height was lowered to 2.90 m and the three-point line was replaced by a rectangle located 4 m from the basket. Plyometric variables, such as number of impacts (total and in zones), number of horizontal impacts (total and in zones), number of steps, number of jumps (total and in zones) and g-force of jumps during takeoff and landing, were assessed using GPS monitoring. In addition, the moderating effect of maturational age on the intervention in each of the variables under study will be evaluated. The results showed that the modified tournament (MT) showed significant differences compared to the standard format (FEB) in playing time, steps, landings 5–8 G, and takeoffs >8 G during positional attacks, as well as in horizontal impact variables during counterattacks and effective playing time. Bayesian analysis provided moderate-to-strong evidence for several of these variables, and extreme evidence for playing time and impacts during effective time. Moreover, maturational age (%PAH) consistently moderated the intervention effects, particularly in impact loads and locomotor demands. These findings can provide useful insights for coaches and practitioners in youth basketball. Adjusting competition rules and considering maturational status may optimize player development by creating contexts that enhance plyometric performance while adapting to the physical and biological characteristics of young athletes.

## 1. Introduction

Youth basketball development presents unique challenges due to the rapid biological, neuromuscular, and motor maturation that occurs during preadolescence [1]. During this stage, players experience substantial changes in height, body composition, and muscular strength, all of which influence performance outcomes in sport-specific tasks [2]. Preadolescence is also characterized by high inter-individual variability, with early and late matures demonstrating markedly different physical capacities and neuromuscular coordination [3]. This variability presents a challenge for coaches, as standardized training or competition formats may not equally benefit all players, potentially limiting skill development or increasing injury risk [4]. Plyometric performance is a fundamental determinant of basketball success, encompassing vertical and horizontal jumping ability, rapid force production, and coordinated landings [5]. These abilities underpin crucial game actions, including shooting, rebounding, sprinting, and defensive maneuvers, and are closely linked to agility and change-of-direction speed [6,7]. Plyometric exercises enhance the stretch-shortening cycle of muscles, improving neuromuscular efficiency and the ability to tolerate high-impact forces commonly encountered during competitive play [8]. Most studies examining plyometric development have focused on structured training programs, with less attention paid to the natural stimuli provided by competition. Competitive basketball matches are complex, high-intensity, and intermittent, requiring frequent jumps, accelerations, decelerations, and changes of direction [9]. The format of competition, including rules, court dimensions, and scoring systems, may influence the type and frequency of these movements, thereby affecting the overall plyometric load experienced by young players [10]. For example, lowering the basket or modifying the three-point line may encourage more dynamic movements, creating different neuromuscular demands compared to standard formats [11]. However, despite the relevance of competition design, the existing literature provides limited evidence on how modified competition formats influence physical load and plyometric demands in youth basketball. While previous studies have examined physical demands across categories or small-sided games [9,10], few have assessed competition formats that intentionally adapt rules for developmental purposes. Likewise, although biological maturation is recognized as a key determinant of performance [2,3], its role has rarely been examined as a continuous moderating variable within modified competition contexts. This constitutes a clear gap in the literature. Biological maturation significantly affects the response to these physical demands. Early maturing players tend to outperform their peers in strength, speed, and jumping ability, allowing them to execute more high-intensity actions during competition [2,3]. Consequently, maturation should be considered when evaluating performance outcomes or designing competition formats, as it moderates the exposure to physical stimuli and may influence skill acquisition and long-term development [4,12]. Adjusting competition rules has been suggested as a strategy to optimize physical and technical development in youth basketball. Modifications such as reduced basket height, altered scoring lines, or adapted game durations can increase engagement, encourage more frequent high-intensity actions, and better match the physical capacities of players at different stages of maturation [13,14]. These adaptations may provide a practical means to create developmentally appropriate competitive environments that challenge players physically while promoting technical and tactical growth. Despite these theoretical benefits, the empirical evidence available is limited. Prior work has examined differences between competition models [11], but these studies have not fully explored the maturation-related disparities that may arise, nor have they evaluated whether modified formats can reduce such inequalities. Additionally, earlier studies often treat maturity in categorical terms (e.g., early vs. late matures), potentially overlooking more nuanced moderating effects derived from continuous modelling approaches. Addressing these limitations is essential to better understand how competition design can support equitable and developmentally aligned participation.

Therefore, this study contributes to the existing literature by examining, for the first time, how two U13 basketball competition formats—official and modified—affect plyometric performance while modelling biological maturity as a continuous moderator. This approach allows for a more precise analysis of how load and performance differences emerge across the maturity spectrum.

The aim of this study was to analyse plyometric performance across two basketball competition models in under-13 players. The authors hypothesize that the modified tournament reduces disparities in plyometric load between players with different maturity levels, thereby offering a competition environment more aligned with the developmental needs of preadolescent athletes. This hypothesis is consistent with the moderation-based analytical strategy employed in the study.

## 2. Materials and Methods

### 2.1. Participants

Forty-one under-13 male basketball players (mean age = 12.91 ± 0.57 years) from four teams in southeastern Spain were initially recruited. As selection criteria, players from the teams available during the data collection period were chosen. These players had to belong to federated teams, train three times per week, and participate in one competitive match. In addition, they were required to have a minimum of one year of playing experience. All were expected to complete pre-tournament assessments and participate in both the official and modified tournaments. Four players did not meet these requirements and were excluded, resulting in a final sample of 37 participants (*n* = 37). This sample size is consistent with previous studies in this field. All participants signed the informed consent form, and the study was approved by the Institutional Research Ethics Committee of the University of Murcia (No. 2828/2020).

### 2.2. Design

The study consisted of two competitive tournaments held over a single weekend. Each team played three matches per tournament following the same competitive structure (round-robin format, identical scheduling, and standardized warm-up procedures). This design ensured that performance comparisons between conditions were not influenced by differences in match order, rest time, or contextual factors.

On the first day, all games were conducted under the official under-14 rules of the FIBA. All players listed on the scoresheet were required to participate in at least one quarter within the first three, with a maximum of two consecutive quarters played. Substitutions were only allowed in cases of injury or disqualification due to five personal fouls. Match duration, court dimensions, ball size, and officiating criteria followed standard FEB guidelines.

On the second day, a tournament using modified rules (MT) was implemented. Basket height was reduced to 2.90 m, and the three-point area used in Spanish mini-basket competitions—a rectangular zone positioned 4 m from the basket—was adopted. Field goals attempted from beyond this line were awarded 3 points, while shots taken from the official 6.75 m distance were awarded 4 points. This rule set was selected because it represents an intermediate stage between the regulations applied at earlier developmental levels and those used in senior competition.

Across both days, teams were required to maintain the same roster and player rotation guidelines, and all matches were officiated by licensed referees. Warm-up duration, rest intervals between games, and data-collection procedures (tracking, performance indicators, and contextual information) were standardized to ensure comparability between competitive conditions. This regulation was chosen because it is an intermediate regulation between the preliminary stages and senior sport.

### 2.3. Procedure and Materials

In each tournament, the following kinematic data were collected: (a) number of impacts (total and in zones); (b) number of horizontal impacts (total and in zones), (c) number of steps, (d) number of jumps (total and in zones) and (e) g-force of jumps during takeoff and (f) landing. Measures were gathered using a real-time motion tracking system that includes a local positioning system (LPS) device based on UWB technology and an inertial measurement unit (IMU; WIMU PROTM, RealTrack Systems, Almeria, Spain) in an indoor basketball court. For data extraction, the software was SPRO version 989 (RealTrack Systems, Almeria, Spain). This instrument was validated in a previous study [15]. Before the tournament, somatic maturation data were collected. Specifically, players’ body mass and standing height were measured, together with the height of both parents, using a Tanita stadiometer (Tanita BF-522W, Tokyo, Japan). All measurements were conducted in accordance with the standards set by the International Society for the Advancement of Kinanthropometry (ISAK) and were carried out by the same researcher, who is certified at ISAK Level 1. In addition, the date of birth and the date of the intervention were recorded. Based on these variables, maturational age was estimated through the percentage of predicted adult height (%PAH), calculated using the Khamis–Roche method [16,17]. This continuous %PAH variable was subsequently used to analyse how somatic maturation moderated the intervention.

**Figure 1:**
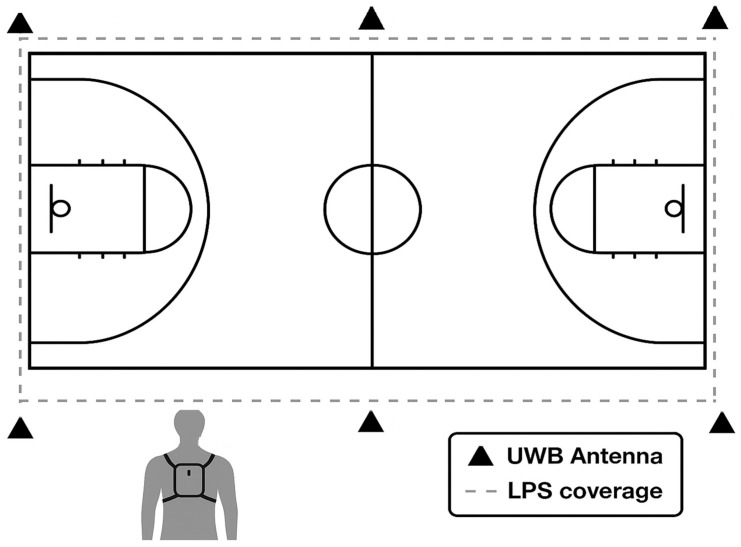
Representation of the location of the UWB antennas.

To ensure the accuracy and reliability of data acquisition, all measurement systems were positioned and configured according to the manufacturer’s recommendations and prior validation protocols. LPS (UWB-based) antennas were installed around the perimeter of the indoor court in a symmetrical rectangular configuration at a height of 3–4 m, ensuring complete coverage of the playing area and minimizing signal shadowing or occlusion. Each player wore the WIMU PRO™ device attached to a snug harness positioned between the shoulder blades. Data sampling rates were set to 1000 Hz for the IMU’s inertial signals. To enhance reliability, all devices were powered on simultaneously, battery levels were checked before each match, and players were instructed not to adjust or tamper with the units during play. Figure 1 presents a diagram illustrating the antenna positions, the player-carrying unit, and the overall tracking setup to facilitate reproducibility and understanding of the instrumentation configuration.

### 2.4. Data Analysis

The normality of data distributions was assessed using the Shapiro–Wilk test. For variables meeting the assumption of normality, paired-samples Student’s t-tests were conducted to compare the two tournaments. When the assumption of normality was violated, the non-parametric Wilcoxon signed-rank test was used (the non-parametric variables were marked with a *). Effect size was calculated as Cohen’s d for normal paired variables and rank biserial correlation for Wilcoxon tests.

In addition, to complement the frequentist approach and to strengthen inference in a relatively small sample, a Bayesian paired-samples t-test was performed. The Bayesian framework allows quantification of the strength of evidence in favour of the alternative (H_1_) or the null (H_0_) hypothesis, rather than relying solely on significance thresholds. The Bayes Factor (BF_10_) was used for interpretation, as it directly indicates how much more likely the data are under H_1_ than under H_0_ [18]. The following interpretive scale (Table 1) was applied: <1/100 = extreme evidence for H_0_; 1/100–<1/30 = very strong for H_0_; 1/30–<1/10 = strong for H_0_; 1/10–<1/3 = moderate for H_0_; 1/3–<1 = ambiguous for H_0_; 1–3 = anecdotal for H_1_; >3–10 = moderate for H_1_; >10–30 = strong for H_1_; >30–100 = very strong for H_1_; >100 = extreme for H_1_ [17].

A within-subject repeated-measures moderation analysis was conducted to examine whether players’ maturational age influenced the effect of the rule change (differences between tournaments). The relationship between the predictor variable and the outcome variable was assessed by testing their interaction with the continuous moderator variable (W1) using a simple moderation model [19]. To this end, an ordinary least squares regression analysis was performed with the SPSS macro MEMORE v2.1 [19]. Given the continuous nature of all variables, the Johnson–Neyman technique was applied to identify regions of significance—specific ranges of maturational age at which the intervention produced statistically significant effects [20]. This statistical procedure was used due to it allowed us to identify the exact regions where significance appears. Using age of maturity as a categorical variable is less precise than this method.

For all analyses, the significance level was set at α = 0.05. Alongside *p*-values, confidence intervals and effect sizes were reported to facilitate interpretation of the magnitude and practical relevance of the effects observed.

## 3. Results

Table 2 presents the differences between the standard tournament and the modified tournament during positional attack phases, in terms of playing time, number of steps, and number of jumps.

The data show statistically significant differences for the variables Playing time (*p* = 0.008; d = 0.462), Steps (*p* = 0.013; d= 0.432), Landing 5–8 G (*p* = 0.008; IQR= 0.559), and Takeoff >8 G (*p* = 0.006; d = 1), with a moderate BF10 observed for these measures.

**Figure 2:**
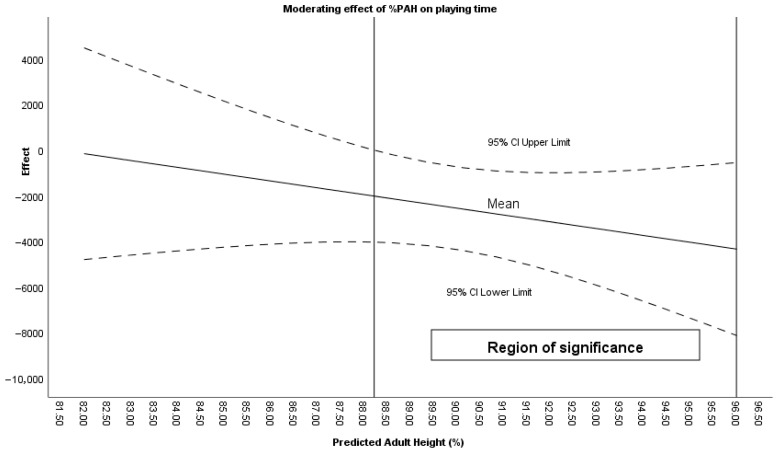
Moderating effect of %PAH on playing time during positional attacks.

The Johnson–Neyman procedure identified ranges in which maturational age (%PAH) moderated the effect of the intervention. For the variable Playing time, a maturational development greater than 88.22% PAH conditioned the intervention effect (Figure 2). For Steps, the effect was conditioned by a %PAH between 87.99% and 93.90%. For Landing 5–8 G, the intervention was conditioned by a %PAH between 86.43% and 93.75%. Finally, for Takeoff >8 G, the intervention effect was conditioned by a %PAH between 86.16% and 92.13%.

Table 3 shows the differences between the standard tournament and the modified tournament during positional attack phases in impact-related variables.

Significant differences were found for total impacts (*p* = 0.025; d = 0.383), impacts between 0–3 G (*p* = 0.028; d = 0.376), impacts between 3–5 G (*p* = 0.029; d = 0.373), horizontal impacts (*p* = 0.025; d = 0.385), horizontal impacts between 0–3 G (*p* = 0.028; d = 0.376), and horizontal impacts between 3–5 G. However, the Bayes factor indicated that these differences were anecdotal.

**Figure 3:**
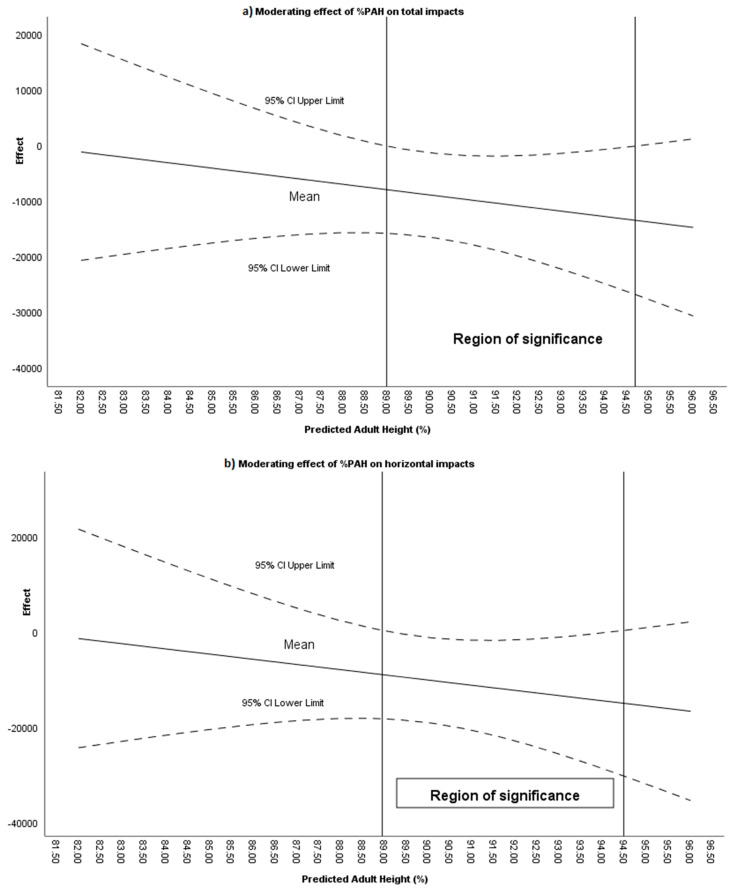
(**a**) Moderating effect of %PAH on total impacts during positional attacks. (**b**) Moderating effect of %PAH on horizontal impacts during positional attacks.

Maturational age, expressed as %PAH, was found to moderate the intervention effect in the following variables: total impacts (88.99–94.68%; Figure 3a), impacts between 0–3 G (89.11–94.49%), impacts between 3–5 G (88.86–92.40%), horizontal impacts (88.95–94.48%; Figure 3b), horizontal impacts between 0–3 G (89.02–94.03%), and horizontal impacts between 3–5 G (>89.66%).

Table 4 presents the differences in the counterattack phase for counterattack time, steps, and jumps.

Significant differences were found for Steps (*p* = 0.020; d = 0.400), Landing 0–3 G (*p* = 0.006; IQR = 0.1), and Takeoff >8 G (*p* = 0.052; IQR = 0.672). The Bayes factor indicated that the differences were anecdotal for Steps and Takeoff >8 G, strong for Landing 0–3 G, and extreme for Landing 3–5 G.

**Figure 4:**
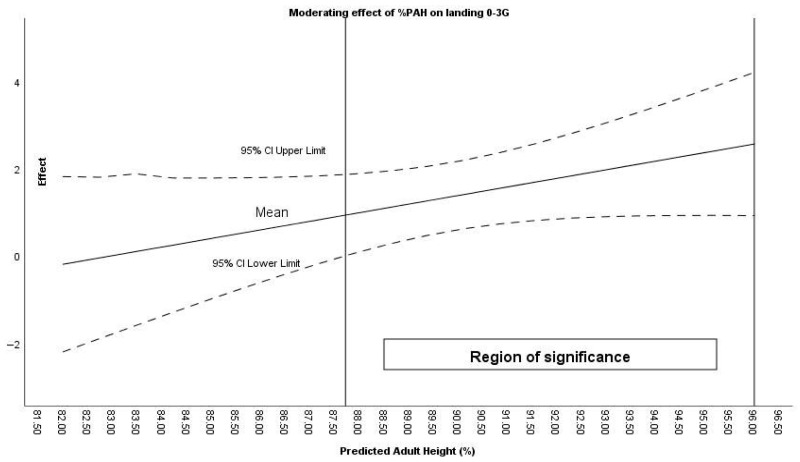
Moderating effect of %PAH on landing 0–3 G during counterattacks.

Maturational age (%PAH) moderated the effect of the intervention in steps, landing 0–3 G and takeoff >8 G. Specifically, the effect was significant for steps when %PAH was greater than 89.08%, for landing 0–3 G above 87.72% (Figure 4), and for takeoff >8 G above 89.67%.

Table 5 shows the differences observed during the counterattack phase for impact-related variables.

Significant differences were found for impacts 0–3 G (*p* = 0.027; d = 0.379), horizontal impacts (*p* = 0.019; d = 0.402), horizontal impacts 0–3 G (*p* = 0.023; d = 0.394), and horizontal impacts 3–5 G (*p* = 0.002; d = 0.554). The effect size was anecdotal for impacts 0–3 G, horizontal impacts, and horizontal impacts 0–3 G, whereas it was strong for horizontal impacts 3–5 G.

**Figure 5:**
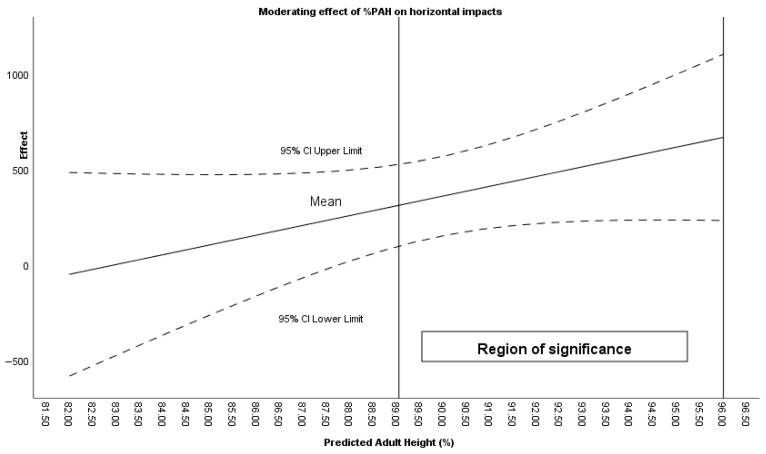
Moderating effect of %PAH on horizontal impacts during counterattacks.

%PAH was found to moderate the effect of the intervention on total impacts (%PAH > 89.19), horizontal impacts (%PAH > 89.05) (Figure 5), horizontal impacts 0–3 G (%PAH > 89.15), and horizontal impacts 3–5 G (%PAH > 87.76).

Table 6 presents the variables of playing time, steps, and jumps during effective playing time.

Significant differences were found for playing time (*p* = 0.002; d = 0.535), steps (*p* = 0.006; d = 0.483), jumps (*p* = 0.019; d = 0.405), Landing 5–8 G (*p* = 0.018; IQR = 0.479), Takeoff 0–3 G (*p* = 0.012; d = 0.435), and Takeoff >8 G (*p* = 0.005; IQR = 0.754). Bayesian analysis indicated strong evidence for playing time and moderate evidence for steps, Takeoff 0–3 G, and Takeoff > 8 G.

**Figure 6:**
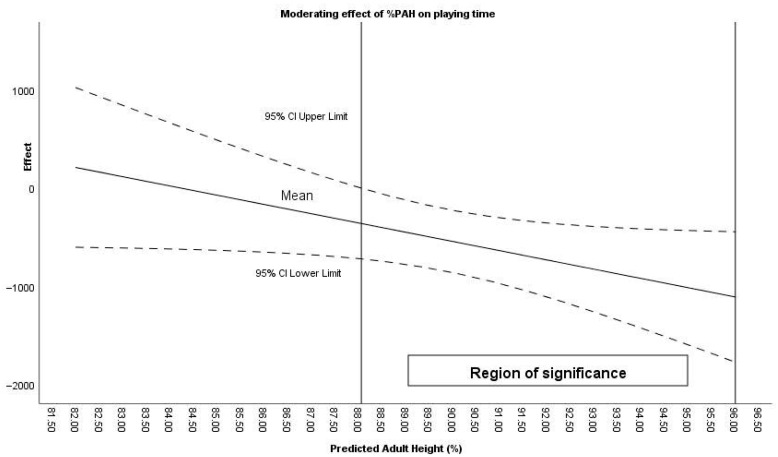
Moderating effect of %PAH on playing time during effective time.

Moderator analysis using the Johnson–Neyman procedure identified that %PAH moderated the effect of the intervention on playing time (%PAH > 88.06) (Figure 6), steps (%PAH > 88.41), jumps (%PAH > 88.97), Landing 5–8 G (%PAH between 88.45 and 94.80), and Takeoff >8 G (%PAH between 86.69 and 93.17).

Table 7 reflects the differences in impacts during effective playing time.

**Figure 7:**
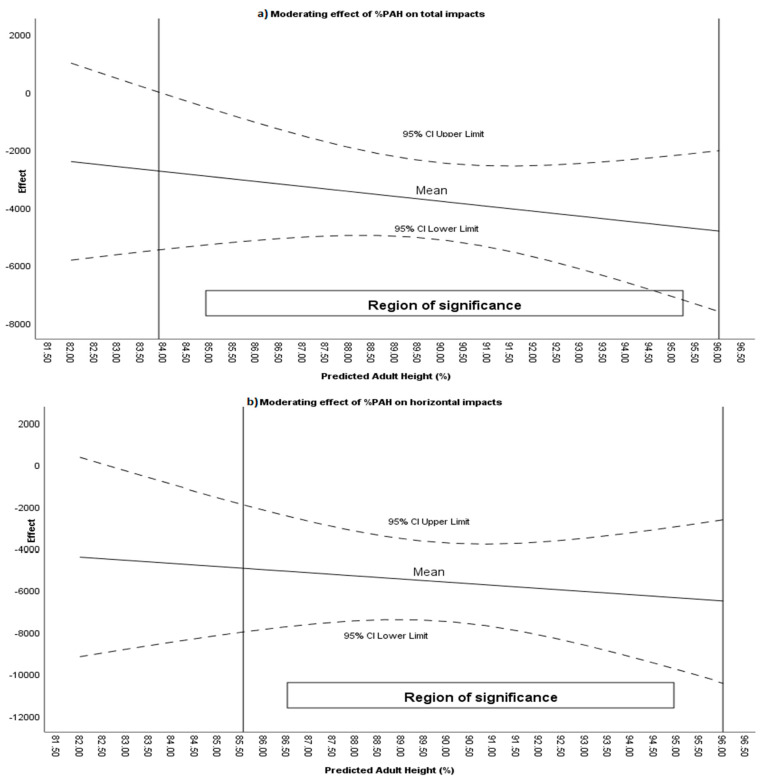
(**a**) Moderating effect of %PAH on impacts during effective time. (**b**) Moderating effect of %PAH on horizontal impacts during effective time.

Statistically significant differences were found for all variables except Impacts >8 G (n). The Bayes factor indicated anecdotal evidence for Horizontal Impacts 3–5 G (n) and Horizontal Impacts 5–8 G (n), moderate evidence for Horizontal Impacts 0–3 G and Horizontal Impacts > 8 G (n), and extreme evidence for total impacts, Impacts 0–3 G, Impacts 3–5 G, Impacts 5–8 G, and horizontal impacts. Moderator analysis using the Johnson–Neyman procedure identified that %PAH moderated the effect of the intervention total impacts (%PAH > 83.89) (Figure 7); impacts 0–3 G (%PAH > 83.86); impacts 3–5 G (%PAH > 84.56); impacts 5–8 G (%PAH > 85.81); horizontal impacts (%PAH > 85.55) horizontal impacts 0–3 G (%PAH > 88.41); horizontal impacts 3–5 G (%PAH > 88.96); horizontal impacts 5–8 (%PAH > 88.91); and horizontal impacts > 8 (%PAH > 88.33).

In general terms, the results show that the Modified Tournament did not affect all variables uniformly. On the one hand, the disparity was reduced in variables associated with overall load, such as the total number of impacts or the number of steps during effective playing time, where inter-individual differences tended to narrow. However, the disparity clearly increased in variables related to high-magnitude plyometric load, such as landings in the 5–8 G range and especially take-offs above 8 G. This increase in variability was modulated by biological maturation, as more mature players showed much greater increases in playing time, horizontal impacts, and high-power actions.

## 4. Discussion

The findings of this study indicate that rule modifications in under-13 basketball players significant increases in plyometric-related load, such as increased steps, landings in the 5–8 G range, and takeoffs over 8 G, particularly during effective playing time. These results align with prior research demonstrating how adapted competitive formats can alter physical and neuromuscular demands in young athletes, creating more dynamic environments that suit their biological development [4,5]. Biological maturation (%PAH) emerged as a key moderator: more mature players showed greater increases in playing time, horizontal impacts, and high-magnitude takeoffs. In that way, the scientific evidence that those who mature early tend to have advantages in strength, power, and speed, enabling them to tolerate higher physical loads in both training and competition [4,21]. However, while early matures may display superior physical metrics, this can mask gaps in technical or tactical skills during early stages, suggesting the need for competition designs that provide equitable developmental opportunities regardless of maturation speed [1].

The observed increases in total impacts and mechanical load under rule-modified tournaments have implications both for neuromuscular adaptation and injury risk management. Prior studies have shown that well-designed plyometric training improves stretch-shortening cycle efficiency, enhances rapid force production, and improves landing control [5,6]. However, excessive or poorly regulated exposure to high-impact movements in youth competition can elevate the risk of overuse injuries, especially in players undergoing rapid growth spurts.

From an applied perspective, modifying competition rules (e.g., lowering basket height, adjusting the three-point line, changing scoring) appears effective in increasing physical stimulus frequency and intensity. These modifications not only make the game more engaging and physically demanding in appropriate ways, but also encourage technical and tactical growth, in line with recent pedagogical arguments for youth sport that emphasize decision making, creativity, and skill development [21,22].

Taken together, this study adds empirical support to the importance of considering biological maturation in youth basketball competition design. Adjusting competition formats can help level the exposure to physical and technical stimuli, thereby promoting more equitable development across players with differing maturation rates, as is the case in other sports [11].

## 5. Conclusions

This study shows that rule modifications in U13 basketball increase players’ exposure to plyometric actions, particularly steps, landings, and high-intensity takeoffs. Moreover, biological maturation (%PAH) influences the magnitude of these effects, with more mature players assuming greater mechanical loads. Overall, adapting competition rules appears to be an effective strategy to provide appropriate physical and developmental stimuli while accounting for individual differences in maturation.

Future research should extend these findings to include longer competitive periods, measures of technical-tactical variables (decision-making, perception), psychological aspects (motivation, enjoyment), and explicit injury surveillance, to better understand the long-term effects of competitive rule modifications. The study has some limitations, most notably the duration of the intervention. Therefore, it is necessary to explore the long-term effects of this intervention. Similarly, the subjects studied are young players, so it is necessary to analyze how it affects older and more experienced players.

## Figures and Tables

**Table 1:** Interpretive Scale for Bayes Factor (BF_10_).

Bayes Factor (BF_10_)	Interpretation	Favours
<1/100	Extreme evidence	H_0_
1/100–<1/30	Very strong evidence	H_0_
1/30–<1/10	Strong evidence	H_0_
1/10–<1/3	Moderate evidence	H_0_
1/3–<1	Ambiguous evidence	H_0_
1–3	Anecdotal evidence	H_1_
>3–10	Moderate evidence	H_1_
>10–30	Strong evidence	H_1_
>30–100	Very strong evidence	H_1_
>100	Extreme evidence	H_1_

**Table 2:** Differences between tournaments in positional attacks for playing time, steps, and jumps.

	Mean ± SD		*p*-Value	Effect Size	Bayesian Factor (BF_10_)	
	Standard Tournament	Modified Tournament				
Playing time (s)	1952 ± 496	2202 ± 569	0.008	0.462	5.06	Moderate
Steps (n)	3449.13 ± 852.50	3896.70 ± 1131.53	0.013	0.432	3.46	Moderate
Jumps (n) *	16.22 ± 7.76	19.32 ± 11.94	0.100	0.312	1.06	Anecdotal
Landing 0–3 G (n)	0	0	-	-	-	-
Landing 3–5 G (n) *	11.46 ± 5.17	12.38 ± 7.93	0.585	0.111	0.235	No evidence
Landing 5–8 G (n) *	4.03 ± 3.18	5.78 ± 4.28	0.008	0.559	8.78	Moderate
Landing > 8 G (n) *	0.73 ± 1.10	1.16 ± 1.44	0.180	0.333	0.510	No evidence
Takeoff 0–3 G (n)	11.00 ± 5.48	12.73 ± 7.56	0.196	0.245	0.698	No evidence
Takeoff 3–5 G (n)	3.81 ± 2.54	4.41 ± 3.94	0.473	0.154	0.297	No evidence
Takeoff 5–8 G (n)	1.27 ± 1.97	1.70 ± 1.71	0.131	0.320	0.452	No evidence
Takeoff > 8 G (n)	0.14 ± 0.42	0.49 ± 0.90	0.006	1.000	4.08	Moderate

* Significant difference (*p* < 0.05).

**Table 3:** Differences between tournaments in positional attacks for impact-related variables.

	Mean ± SD		*p*-Value	Effect Size	Bayesian Factor (BF_10_)	
	Standard Tournament	Modified Tournament				
Total impacts (n)	8117.05 ± 2021.09	8988.54 ± 2274.15	0.025	0.383	1.92	Anecdotal
Impacts 0–3 G (n)	7079.65 ± 1899.87	7841.08 ± 2027.51	0.028	0.376	1.75	Anecdotal
Impacts 3–5 G (n)	799 ± 221.30	862.838 ± 273.379	0.029	0.373	1.70	Anecdotal
Impacts 5–8 G (n) *	229.54 ± 86.53	252.027 ±119.982	0.064	0.356	0.774	No evidence
Impacts * >8 G (n) *	28.87± 18.70	32.595 ± 28.377	0.432	0.149	0.375	No evidence
Horizontal Impacts (n)	10,401.24 ± 2569.41	11,443.65 ± 3054.89	0.025	0.385	1.94	Anecdotal
Horizontal Impacts 0–3 G (n)	10,171 ± 2542	11,180 ± 3025	0.028	0.376	1.75	Anecdotal
Horizontal Impacts 3–5 G (n) *	204.03 ± 98.46	229.89 ± 136.88	0.065	0.350	1.23	Anecdotal
Horizontal Impacts 5–8 G (n) *	22.78 ± 16.04	29.84 ± 33.38	0.171	0.263	0.823	No evidence
Horizontal Impacts > 8 G (n) *	2.95 ± 3.42	4.22 ± 5.80	0.215	0.279	0.509	No evidence

* Significant difference (*p* < 0.05).

**Table 4:** Differences between tournaments in counterattacks for playing time, steps, and jumps.

	Mean ± SD		*p*-Value	Effect Size	Bayesian Factor (BF_10_)	
	Standard Tournament	Modified Tournament				
Playing time (s)	1478.24 ± 429.91	1599.14 ± 466.99	0.107	0.272	0.610	No evidence
Steps (n)	2894.81 ± 908.17	3245.57 ± 925.34	0.020	0.400	2.34	Anecdotal
Jumps (n) *	14.5 ± 9.33	13.43 ± 8.65	0.333	0.186	0.304	No evidence
Landing 0–3 G (n) *	1.351 ± 2.41	0 ± 0	0.006	1	20.73	Strong
Landing 3–5 G (n)	7.30 ± 4.52	8.49 ± 5.28	<0.001	2.66	1.18 × 10^15^	Extreme
Landing 5–8 G (n) *	4.43 ± 3.45	3.73 ± 3.45	0.146	0.303	0.652	No evidence
Landing > 8 G (n) *	1.22 ± 1.67	1.41 ± 2.71	0.771	0.064	0.206	No evidence
Takeoff 0–3 G (n) *	6.70 ± 4.73	7.33 ± 4.74	0.306	0.208	0.295	No evidence
Takeoff 3–5 G (n) *	4.30 ± 3.46	3.70 ± 2.91	0.221	0.252	0.395	No evidence
Takeoff 5–8 G (n) *	1.92 ± 2.24	1.97 ± 2.09	0.898	0.031	0.179	No evidence
Takeoff > 8 G (n) *	0.21 ± 0.48	0.43 ± 0.84	0.052	0.672	1.23	Anecdotal

* Significant difference (*p* < 0.05).

**Table 5:** Differences between tournaments in counterattacks for impact-related variables.

	Mean ± SD		*p*-Value	Effect Size	Bayesian Factor (BF_10_)	
	Standard Tournament	Modified Tournament				
Total impacts (n)	6257.62 ± 1770.60	6782.68 ± 1818.56	0.099	0.278	0.646	No evidence
Impacts 0–3 G (n)	4965.62 ± 1666.56	5584.73 ± 1515.10	0.027	0.379	1.82	Anecdotal
Impacts 3–5 G (n) *	953.46 ± 388.70	8668.70 ± 326.34	0.420	0.154	0.360	No evidence
Impacts 5–8 G (n) *	293.03 ± 112.17	288.76 ± 139.72	0.803	<0.001	0.744	No evidence
Impacts > 8 G (n) *	45.57 ± 29.23	40.49 ± 33.79	0.164	0.267	0.355	No evidence
Horizontal Impacts (n)	8765.22 ± 2721.83	9866.43 ± 2788.66	0.019	0.402	2.38	Anecdotal
Horizontal Impacts 0–3 G (n)	8542.62 ± 2681.27	9602.054 ± 2747.281	0.023	0.394	2.10	Anecdotal
Horizontal Impacts 3–5 G (n)	578.30 ± 622.24	224.76 ± 137.99	0.002	0.554	18.41	Strong
Horizontal Impacts 5–8 G (n) *	36.32 ± 31.25	35.43 ± 34.65	0.670	0.084	0.181	No evidence
Horizontal Impacts > 8 G (n) *	4.22 ± 5.14	4.19 ± 4.81	0.888	0.030	0.177	No evidence

* Significant difference (*p* < 0.05).

**Table 6:** Differences between tournaments in effective playing time for playing time, steps, and jumps.

	Mean ± SD		*p*-Value	Effect Size	Bayesian Factor (BF_10_)	
	Standard Tournament	Modified Tournament				
Playing time (s)	3012.35 ± 656.96	3542.00 ± 967.44	0.002	0.535	14.10	Strong
Steps (n)	6088.70 ± 1339.55	6927.19 ± 1783.53	0.006	0.483	6.73	Moderate
Jumps (n)	27.92 ± 14.67	32.92 ± 19.70	0.019	0.405	2.49	Anecdotal
Landing 0–3 G (n) *	0.19 ± 0.57	0 ± 0	0.048	1	1.09	Anecdotal
Landing 3–5 G (n) *	17.95 ± 8.52	21.05 ± 11.94	0.058	0.359	1.04	Anecdotal
Landing 5–8 G (n) *	7.87 ± 5.45	9.514 ± 7.20	0.018	0.479	2.87	Anecdotal
Landing > 8 G (n) *	2.08 ± 3.29	2.351 ± 2.94	0.581	0.125	0.230	No evidence
Takeoff 0–3 G (n)	16.68 ± 8.10	20.108 ± 11.40	0.012	0.435	3.57	Moderate
Takeoff 3–5 G (n) *	7.60 ± 5.02	8.378 ± 6.44	0.313	0.206	0.299	No evidence
Takeoff 5–8 G (n) *	0.38 ± 0.72	0.919 ± 1.36	0.717	0.078	0.204	No evidence
Takeoff > 8 G (n) *	3.30 ± 3.56	3.514 ± 3.40	0.005	0.745	5.83	Moderate

* Significant difference (*p* < 0.05).

**Table 7:** Differences between tournaments in effective playing time for impact-related variables.

	Mean ± SD		*p*-Value	Effect Size	Bayesian Factor (BF_10_)	
	Standard Tournament	Modified Tournament				
Total impacts (n) *	10,771.38 ± 4242.79	15,242.00 ± 3564.7	<0.001	0.829	51,918.31	Extreme
Impacts 0–3 G (n)	9860.65 ± 2699.78	13,095.19 ± 3162.24	<0.001	0.939	10,555.94	Extreme
Impacts 3–5 G (n) *	1309.35 ± 417.54	1703.46 ± 557.99	<0.001	0.749	1664.87	Extreme
Impacts 5–8 G (n)	400.30 ± 174.04	533.32 ± 250.64	<0.001	0.806	1075.16	Extreme
Impacts >8 G (n) *	68.8 ± 47.6	86.4 ± 75.6	0.762	0.056	0.442	No evidence
Horizontal Impacts (n)	15,278.38 ± 4376.97	20,875.27 ± 5268.13	<0.001	1.01	39,064.16	Extreme
Horizontal Impacts 0–3 G (n)	17,884.78 ± 4053.28	20,354.38 ± 5225.91	0.006	0.476	6.15	Moderate
Horizontal Impacts 3–5 G (n) *	384.57 ± 196.38	448.08 ± 273.52	0.009	0.496	2.76	Anecdotal
Horizontal Impacts 5–8 G (n) *	47.51 ± 38.99	64.38 ± 66.97	0.003	0.564	2.95	Anecdotal
Horizontal Impacts > 8 G (n) *	5.12 ± 6.27	8.43 ± 9.70	0.003	0.604	3.91	Moderate

* Significant difference (*p* < 0.05).

## Data Availability

Data are contained within the article.
